# Assessment of Carbon Nanotubes on Barrier Function, Ciliary Beating Frequency and Cytokine Release in In Vitro Models of the Respiratory Tract

**DOI:** 10.3390/nano13040682

**Published:** 2023-02-09

**Authors:** Claudia Meindl, Markus Absenger-Novak, Ramona Jeitler, Eva Roblegg, Eleonore Fröhlich

**Affiliations:** 1 Center for Medical Research, Medical University of Graz, Stiftingtalstr. 24, 8010 Graz, Austria; 2 Department of Pharmaceutical Technology and Biopharmacy, Institute of Pharmaceutical Sciences, University of Graz, Universitaetsplatz 1, 8010 Graz, Austria

**Keywords:** carbon nanotubes, toxicity, in vitro models, respiratory tract, bronchial epithelium, alveolar epithelium, ciliary beating frequency

## Abstract

The exposure to inhaled carbon nanotubes (CNT) may have adverse effects on workers upon chronic exposure. In order to assess the toxicity of inhaled nanoparticles in a physiologically relevant manner, an air–liquid interface culture of mono and cocultures of respiratory cells and assessment in reconstructed bronchial and alveolar tissues was used. The effect of CNT4003 reference particles applied in simulated lung fluid was studied in bronchial (Calu-3 cells, EpiAirway™ and MucilAir™ tissues) and alveolar (A549 +/−THP-1 and EpiAlveolar™ +/−THP-1) models. Cytotoxicity, transepithelial electrical resistance, interleukin 6 and 8 secretion, mucociliary clearance and ciliary beating frequency were used as readout parameters. With the exception of increased secretion of interleukin 6 in the EpiAlveolar™ tissues, no adverse effects of CNT4003 particles, applied at doses corresponding to the maximum estimated lifetime exposure of workers, in the bronchial and alveolar models were noted, suggesting no marked differences between the models. Since the doses for whole-life exposure were applied over a shorter time, it is not clear if the interleukin 6 increase in the EpiAlveolar™ tissues has physiological relevance.

## 1. Introduction

Nanoparticles are used in industry, consumer products, health care and medicine, but the safety of some particles is still a matter of discussion [1]. One particle type with unclear safety is carbon nanotubes (CNTs). CNTs have been evaluated for cytotoxicity, genotoxicity, inflammatory potential and carcinogenicity with variable results [2]. The absorption of CNTs through epithelial barriers was found to be low. Among all barriers, the respiratory barrier is the less protected and most permeable. The lung consists of the conducting and the respiratory airways, which have different protection mechanisms and require different models for toxicity testing in vitro [3].

Reported cytotoxicity of multi-walled CNTs (MWCNTs) varied from 5 ng/mL to 10 mg/mL and based on a systematic review, A549 and HUVEC were the most appropriate cells for comparison among research groups [4]. The differences in cytotoxicity by a factor of 10^6^ are thought to be due not only to differences in cells, assays, exposure conditions and sample preparation but also to different production of the MWCNTs [4]. To enable better comparison of the results, the use of standardized nanoparticles is suggested. Sponsored by several European Union Joint programs, European Commission’s Directorate General Joint Research Centre (JRC) has established the JRC Nanomaterials Repository of industrially manufactured nanomaterials with the aim of providing the scientific and regulatory communities with nanomaterials for testing [5]. Standard Operation Procedures for the dispersion of titanium dioxide, silicon dioxide and MWCNTs in 0.05% bovine serum albumin (BSA) are available due to the NANOGENOTOX European joint action to exclude differences in the preparation of particle suspensions [6]. The CNT4003 particles, formerly termed NM-403, belong to the thin and short MCNTs with reported primary sizes of 12 nm diameter and 443 nm length. They were chosen for this study because measurements of CNT manufacturing workplaces report sizes of highest exposure between 10 and 100 nm [7,8,9], whereas the thicker and longer CNTs (e.g., 80 nm × 3.7 µm) form particles with aerodynamic size of 260–381 nm [10]. No decrease in viability was observed at 45.7 μg/cm^2^ NM-403 for A549 alveolar epithelial cells in conventional testing [11].

Air–liquid interface culture (ALI), where cells grow on transwell membranes and are exposed to medium with nutrients only from the basal side and the apical parts face the air, is the physiologically relevant culture for respiratory cells. Using this technique, Calu-3 cells, established models for the bronchial epithelium, produce mucus and A549 as models for the alveolar epithelium surfactant [12,13]. Calu-3 cells also form epithelial barriers of sufficient tightness identified by transepithelial electrical resistance (TEER) values of >300 Ω × cm^2^. The limitation of Calu-3 cells is that effects of toxicants on ciliary function, the important clearance mechanism of the upper airways, cannot be assessed because the cells form only immature and not motile cilia [14]. Reconstructed tissues prepared from human bronchial epithelial cells, EpiAirway™ from MatTek Cooperation and MucilAir™ from Epithelix Sárl, on the other hand, possess cilia and, therefore, allow the assessment of this relevant protection system of the lung. The main limitation of A549 cells, which are routinely used in respiratory toxicity testing, is the fact that despite expression of tight junction proteins, they do not form a functional epithelial barrier, which corresponds to the generally low TEER values in the range of 20–60 Ω × cm^2^ [15,16]. EpiAlveolar™ from MatTek is composed of alveolar epithelial cells, fibroblasts and endothelial cells and enables assessment of alveolar barrier function. EpiAlveolar™ enriched with THP-1 macrophages are also available for more realistic testing of the alveolar barrier [17].

To mimic inhalation of MWCNTs, some researchers have used the VITROCELL Cloud system [17,18,19]. According to the producer, 200 µL of nebulization volume are recommended for the 12-well exposure chambers (https://www.vitrocell.com/inhalation-toxicology/exposure-systems/vitrocell-cloud-system/vitrocell-cloud-alpha-6 accessed on 2 May 2021), indicating that each well of a 12-well plate will receive ~17 µL of saline together with the particles. This solution differs in volume and composition from the lung lining fluid in the lung. The surface of the lung is covered by a dipalmitoyl-glycero-3-phosphocholine (DPPC)-rich lung lining fluid of 70–300 nm thickness [20]. The presence of DPPC is important because nanoparticles in contact with biological fluids will absorb biomolecules, proteins and lipids onto their surface [21]. This so called “corona” will determine the cellular response of the host cells. It has been reported in animal studies that coating with DPPC markedly increased uptake and lung retention of nanoparticles compared to coating with BSA [22,23]. To take the effect of the DPPC binding into account, CNT4003 particles were suspended in simulated lung fluid (SLF), a phosphate buffer containing 0.02% DPPC.

Sauer et al. compared the cytotoxicity of respiratory toxicants in A549 and 3T3 monolayers, MucilAir™ and EpiAirway™ tissues [24], but there is no systematic comparison between models based on cell lines and commercially available reconstructed tissues of bronchial and respiratory part. This comparison is important because the use of the more complex reconstructed tissues is associated with higher costs. The aim of this study was to identify adverse effects of standardized CNTs in physiologically relevant systems and to reveal the role of the testing system. To this end, effects of CNT4003 particles were compared in models of different complexity (monolayers vs. multilayers) and origin (primary vs. cell lines). The focus is not on cytotoxicity but on functional aspects because physiologically relevant exposure doses to CNTs are too low to act acutely toxic. For the bronchial part of the airways Calu-3 cells, EpiAirway™ and MucilAir™ were used. Effects of CNT4003 on the alveolar part of the airways were studied using A549 cells and EpiAlveolar™ tissues with and without THP-1 macrophages.

## 2. Materials and Methods

### 2.1. Preparation of Simulated Lung Fluid (SLF)

1,2-Dipalmitoyl-sn-glycero-3-phosphocholine (DPPC, Avanti Polar Lipids, Birmingham, AL, USA) is dissolved in ethanol and added dropwise to prewarmed (37 °C) phosphate-buffered saline with Ca^2+^ and Mg^2+^ (PBS, ThermoFisher Scientific, Vienna, Austria) to reach a concentration of 0.02% (*w*/*v*) DPPC.

### 2.2. Preparation of Particle Suspensions

CNT4003 was obtained from the Joint Research Center (JRC, Ispra, Italy). According to the list of representative nanomaterials status June 2016 of the JRC, CNT4003 are MWCNTs with 189 m^2^/g surface area with average length of 443 nm and average diameter of 12 nm [25]. Suspensions were prepared according to the protocol established in the NANOGENOTOX project [26] in 0.05% (*w*/*v*) BSA and in the same way in SLF to find out if the same protocol works also for SLF. Briefly, 15.36 mg of CNT4003 powder was prewetted with 30 µL absolute ethanol. Subsequently, 0.97 mL of SLF or of 0.05% BSA in distilled water was added while slowly rotating the glass vessel. To prepare the stock solution, additional 5 mL of SLF or of 0.05% (*w*/*v*) BSA was added. Sonication time and amplitude can be optimized, e.g., 16 min at 10% amplitude or 12 min at 20% amplitude can be used [6]. Suspensions were sonicated on ice with a Branson SFX250 Digital Sonifier (Branson Ultrasonic Cooperation, Danbury, USA) equipped with a microtip at 250 W, 20 kHz and 30% amplitude with 10 s impulses (i.e., 10 s pulse on, 1 s pulse off) for 14 min. Pilot experiments showed that the use of a higher amplitude in combination with a shorter sonication time led to a more effective deagglomeration. Suspensions were prepared directly prior to the administration because it was reported that NM-403 showed high polydispersivity and were shown to be stable for 1–2 h.

### 2.3. Determination of Size and Zeta Potential

Measurements were performed at 25 ± 0.5 °C with a particle concentration of 25.6 µg/mL in triplicates. Hydrodynamic size and polydispersity index (PDI) were measured via photon correlation spectroscopy (PCS) using a Zetasizer Nano ZS (Malvern Instruments, Malvern, United Kingdom) equipped with a 532 nm laser. The scattering angle chosen was 173°, and the refractive indices for the CNTs and the dispersion medium were 2.50 and 1.330, respectively. The zeta potential was measured by electrophoretic light scattering (scattering angle of 173°; Zetasizer Nano ZS, Malvern Instruments) considering the same optical properties of the CNTs and the dispersion media. The calculation was based on the electrophoretic mobility using Henry’s equation. The zeta potential was −9.9 ± 0.7 mV for CNT 4003 in BSA and 1.4 ± 0 mV for CNT 4003 in SLF. The hydrodynamic size of particles suspended in 0.05% BSA determined by PCS was 2327 nm with a PDI of 1.0, indicating that PCS was not suitable for accurately determining particle size. In addition, SLF contained DPPS, which formed micelles of 58 ± 7 nm and interfered with the size measurements. Therefore, the sizes of CNT 4003 were additionally measured by laser diffraction (LD, Mastersizer 2000, Malvern Instruments, United Kingdom) by adding the CNT suspension to 18–20 mL SLF or 0.05% BSA until a laser obscuration of 4–6% was achieved. Volume based values considering d (0.1), d(0.5) and d (0.9) were determined in triplicate at 25 °C and CNTs showed d(0.1) 974 ± 32, d(0.5) 2254 ± 44 and d(0.9) 5399 ± 71 nm in BSA and d(0.1) 1290 ± 8, d(0.5) 3829 ± 38 and d(0.9) 6990 ± 43 nm in SLF. These data indicate that the protocol using SLF leads to a particle distribution similar to that observed with BSA.

### 2.4. Cellular Models of Bronchial and Alveolar Epithelium

Calu-3 cells were obtained from LGC Standards GmbH (Wesel, Germany) and cultured in minimum essential medium (MEM), 2 mM L-glutamine, 1% penicillin/streptomycin, 10% fetal bovine serum (FBS) and 1 mM sodium pyruvate. A549 cells obtained from Deutsche Sammlung für Mikroorganismen und Zellkulturen GmbH (Braunschweig, Germany) were cultured in Dulbecco’s modified Eagle’s medium (DMEM), 2 mM L-glutamine, 1% penicillin/streptomycin (P/S) and 10% FBS. THP-1 was purchased from Cell Line Services (Eppelheim, Germany) and cultured in RPMI, 2 mM L-glutamine, 1% penicillin/streptomycin and 10% FBS. Cells were passaged at regular intervals.

For the exposures with the CNTs, 0.5 × 10^6^ Calu-3 cells were seeded in 500 µL MEM, 2% L-glutamine and 1% PS + 10% FBS on 12-well polyethylene terephthalate transwell inserts (pore size 0.4 µm, Greiner Bio-one, Kremsmünster, Austria) with 1500 µL of the same medium in the basolateral compartment. Medium in the apical compartment was removed after 24 h, and the medium amount in the basolateral compartment was reduced to 500 µL, which was changed every 2 or 3 days. Cells were used for the experiments when they had reached a transepithelial electrical resistance (TEER) value of >300 Ω × cm^2^.

A549 was seeded at a density of 0.8 × 10^5^ in DMEM + 10% FBS on 12-well polyethylene terephthalate transwell inserts (pore size 0.4 µm, Greiner Bio-one) with 1500 µL of the same medium in the basolateral compartment. Medium in the apical compartment was removed after 24 h as reported previously [27]. According to these experiments, the ratio of 9 alveolar cells to 1 alveolar macrophage observed in vivo [28] is obtained by seeding THP-1 macrophages to the cultured A549 cells in a ratio 1:1. A549 cells were cultured for 9 days in ALI prior to the addition of the THP-1 cells and the average number of A549 cells was determined in duplicates. For the coculture, RPMI + 10% FBS was added in the basal compartment and the medium changed every other day.

#### Differentiation to THP-1 Macrophages for Coculture with A549 Cells or EpiAlveolar™ Tissues

For coculture with A549 cells, 1.0 × 10^6^ THP-1 cells/mL were seeded in RPMI 1640 containing 10% FBS, 1% L-glutamine and 1% P/S in flasks. Differentiation to macrophages was induced by addition of 10 nM phorbol 12-myristate 13-acetate (PMA, Sigma-Aldrich, Vienna, Austria) to the media for 48 h. The stimulation medium was changed to medium without PMA for another 24 h before use in the cocultures. Cells were harvested by treatment with trypsin/EDTA (0.05%). For addition to EpiAlveolar™ tissues (MatTek Corporation, Ashland, OR, USA), THP-1 cells were stimulated with 8 nM PMA for 72 h, harvested by treatment with trypsin/EDTA and added immediately to the tissues.

### 2.5. Reconstructed Tissues

EpiAirway™ 3D human respiratory epithelial tissues (AIR-100 PC6.5/PE6.5) were obtained from MatTek Corporation. The tissues were cultivated at the air–liquid interface and medium (MatTek Corporation) changed every other day according to the instructions of the producer. EpiAlveolar™ 3D tissues with and without THP-1 macrophages (MatTek Cooperation) were maintained at the air–liquid interface with medium (MatTek Corporation) changes every other day. Since EpiAlveolar™ 3D tissues with THP-1 macrophages did not tolerate the long delivery, they were added to the EpiAlveolar™ 3D tissues in the laboratory in Graz following the protocol of the company in which 25,000 THP-1 macrophages were added per insert. The cells were seeded in 75 µL media into the apical compartment during feeding and after 24 h the culture was switched back to air-liquid interface condition.

MucilAir™ tissues were purchased from Epithelix Sárl (Geneva, Switzerland) and maintained in MucilAir™ culture medium (Epithelix Sárl) in 24-well format Transwell^®^ cell culture inserts (Sigma-Aldrich) in a humidified incubator (37 °C; 5% *v*/*v* CO_2_). The culture medium was changed every 2–3 days.

### 2.6. Exposure to Carbon Nanotubes

CNT4003 in different doses were suspended in SLF. A total of 10 µL of the suspension were used for the 0.336 cm^2^ tissues and 30 µL for the models with 1.12 cm^2^. They were applied on the surface of the cell monolayers or reconstructed tissues. The entire observation time was 14 d. After initial addition of the CNT dose for seven days, cell and tissue surfaces were rinsed with PBS, Transepithelial Electrical Resistance (TEER) measured and new particles or SLF added for another seven days.

### 2.7. Measurement of Transepithelial Electrical Resistance (TEER) Values

TEER values were determined with an EVOM STX-2-electrode (World Precision Instruments, Berlin, Germany). A total of 0.4 mL MEM was added to the apical and 1.2 mL MEM to the basolateral compartment for TEER measurements for EpiAirway™ and 0.5 mL in the apical and 1.5 mL in the basolateral compartment for EpiAlveolar™ tissues. TEER values were calculated as follows: TEER (Ω × cm^2^) = (Sample–blank resistance, given in Ω) xmembrane area, given in cm^2^. Blank resistance is defined as the resistance of the membrane without cells. Membrane area is indicated for EpiAirway™ as 0.336 cm^2^ and for EpiAlveolar™ as 1.12 cm^2^.

### 2.8. Determination of Viability (MTS Assay)

Viability was assessed at the end of the exposure time (14 d). CellTiter 96^®^ AQueous Non-Radioactive Cell Proliferation Assay (Promega, Mannheim, Germany) was used according to the manufacturer’s instructions. To avoid interference with CNT4003 absorption, inserts were first rinsed with cell culture medium. Subsequently, 100 µL of the combined MTS/PMS solution + 500 µL medium was added in each insert. Plates were incubated for up to 2 h at 37 °C in the cell incubator. The supernatant was transferred into a new plate to remove remaining CNT4003 particles and absorbance was read at 490 nm on a plate reader (SPECTRA MAX plus 384, ServoLab, Kumberg, Austria).

### 2.9. Interleukin Measurements

Medium from the basolateral compartments of all cultures was collected, and the re-lease of IL-6 and IL-8 was determined using the human IL-6 ELISA set (BD OptEIA™, BD Biosciences, Heidelberg, Germany) and the human IL-8 ELISA set (BD OptEIA™) according to the protocol given by the producers. To induce IL-6 and IL-8 secretion, 24 h prior to the harvesting, 20 µL containing 1, 5 and 10 µg lipopolysaccharide from Escherichia coli 055:B5 (LPS, Sigma-Aldrich) as proinflammatory stimulus was added. The concentration of LPS, which caused the maximum release of the cytokines by A549 cells was already determined in a previous study [27]. Absorbance was read at 450 nm (with correction wavelength of 570 nm) on a SPECTROstar (ServoLab) photometer.

### 2.10. Mucociliary Clearance

Prior to the measurements, tissues were rinsed in PBS and 10 µL of PBS containing 4 × 10^5^ yellow-green, fluorescent carboxyl polystyrene particles (1.0 µm, Thermo Fisher Scientific, Waltham, MA, USA) was added and migration of the beads monitored for 10–30 s using Ti2-E/confocal system (Nikon CEE GmbH, Vienna, Austria) equipped with heated stage (37 °C) and incubation chamber at 10× magnification. Object tracking was performed by NIS Element software (Nikon CEE GmbH).

### 2.11. Ciliary Beating Frequency

Prior to the measurements, tissues were rinsed in PBS. Tissues were cut out from the insert and stripes of epithelium were placed on a glass slide and put into the incubation chamber (37 °C) on a fully motorized Ti2-E/confocal system (Nikon CEE GmbH) mounted on an antivibration table. Peripheral and central parts of the stripes were imaged. In pilot experiments, propranolol hydrochloride (100 µM, Sigma-Aldrich) was included as inhibitor and 5 µM forskolin (Sigma-Aldrich) + 100 µM 3-Isobutyl-1-methylxanthin (IBMX, Sigma) as stimulator of CBF. Background noise was determined by measurement of samples fixed with 4% paraformaldehyde. The background was negligible at 0.15–0.75 Hz. Further, the reaction to 1, 5 and 10 µg/insert LPS was recorded. Specimens were examined using 20× magnification. Beating ciliated edges were recorded using a digital high-speed video camera (Andor Zyla VSC-08691, Oxford Instruments, Wiesbaden, Germany) at a rate of 100 frames per second with a frame size of 512 × 512 pixel. Five regions of interest (ROI) were analysed per sample. CBF was calculated by NIS Element software (Nikon CEE GmbH) and indicated as beats/second (Hz). In the later experiments, only propranolol was used because no prominent increase in CBF was seen.

### 2.12. Histology

MatTek tissues were fixed in 4% paraformaldehyde and embedded in paraffin using Tissue-Tek^®^VIP™ 5 (SanovaPharma GesmbH, Gallspach, Austria). Radial sections of 2–5 µm were cut at a rotary microtome, stained with hemalaun and viewed with an Olympus BX51 microscope. Additional sections were cut for immunocytochemical staining.

#### 2.12.1. Immunocytochemical Detection of α-Tubulin

Sections were incubated with DakoCytomation Target Retrieval Solution pH 6.0 (Agilent, Vienna Austria) in decloaking chamber (Biocare Medical, Pacheco, CA, USA) for 10 min at 110 °C and 20 s at 85 °C. Sections remained in the solution for cool down for 30 min. Three rinses with PBS were performed prior to the incubations with blocking serum (1% goat serum in PBS for 30 min at RT) and between each staining step. Antibodies were diluted in antibody diluent (DAKOCytomation, Hamburg, Germany). Incubation with anti-α-tubulin antibody (mouse, 1:500, Thermo Fisher Scientific) or control mouse IgG (negative control, Linaris, Mannheim, Germany) for 1 h at RT was followed by incubation with anti-mouse Alexa 488 antibody (goat, 1:400, Thermo Fisher Scientific) in antibody diluent (DAKOCytomation) for 30 min at RT and counterstain with Hoechst 33342 (1 µg/mL, Thermo Fisher Scientific) for 15 min at RT. After three final washes in PBS, sections were mounted with fluorescence mounting media.

#### 2.12.2. Immunocytochemical Detection of Mucin

Antigen retrieval was performed with DakoCytomation Target Retrieval Solution pH 9.0 (DAKO) in decloaking chamber (Biocare Medical) for 10 min at 110 °C and 20 s at 85 °C. Sections remained in the solution for cool down for 30 min. Three rinses with PBS were performed, cells permeabilized with PBS plus 0.2% Triton X-100 for 2 h at RT. Unspecific antibody binding was blocked with 10% normal goat serum for 30 min at RT. Incubation with anti-mucin 5AC (mouse, 1:200, Abcam, Cambridge, United Kingdom) antibody was performed for 1 h at 37 °C, incubation with anti-mouse Alexa 488 antibody (goat, 1:400, Thermo Fisher Scientific) for 1 h at 37 °C and with Hoechst 33342 (1 µg/mL, Thermo Fisher Scientific) in PBS for 15 min at RT.

#### 2.12.3. Immunocytochemical Detection of Macrophages

After deparaffinization, cells were permeabilized by incubation with 0.1% Triton X-100 in PBS for 60 min at RT and incubation with APC-CY7 anti-CD45 (mouse, 1:500, BD Biosciences, Vienna, Austria) for 1 h at RT and counterstain of nuclei with Hoechst 33342 (1 µg/mL, Thermo Fisher Scientific) for 30 min at RT.

#### 2.12.4. Staining of Whole Mounts with Phalloidin

EpiAlveolar™ tissues were fixed for 15 min with 4% paraformaldehyde. After three washes with PBS, the tissues were permeabilized with 0.1%Triton X100 in PBS, washed three times in PBS again and subsequently incubated with Alexa Fluor™ 488 Phalloidin (Thermo Fisher Scientific, 1:100 in antibody diluent) and Hoechst 33342 (1 µg/mL, Thermo Fisher Scientific) for 30 min at RT.

Images were taken at a Ti2-E/confocal system (Nikon CEE GmbH) with ex/em of 395 nm/414–450 nm for Hoechst 33342 and 470 nm/500–530 nm for α-tubulin, mucin 5AC and phalloidin, and 640 nm/660–850 nm for CD45.

### 2.13. Statistics

Experiments were performed in triplicates and repeated at least two times. Data from all experiments were analyzed with one-way analysis of variance (ANOVA) followed by Tukey’s HSD post hoc test for multiple comparisons (SPSS 28 software). Results with *p*-values < 0.05 were considered to be statistically significant.

## 3. Results

In all evaluations, CNT4003 was applied in SLF and the following parameters and assays used as readout: MTS assay for cytotoxicity, TEER measurements for epithelial barrier tightness, interleukin 6 and 8 levels for proinflammatory effects, movement of polystyrene marker particles for mucociliary clearance and high-speed video microscopy for ciliary beating frequency. IL-6 is a marker for acute and chronic inflammation and IL-8 a marker for acute inflammation and a chemoattractant for neutrophils [18]. Doses are indicated as µg/insert and the distribution of CNT4003 on the cell-grown inserts documented. After 14 d of exposure, several CNT4003 agglomerates were visible in the central region of inserts containing mucus-producing cells and rare agglomerates in the inserts containing EpiAlveolar™ tissues (Figure S1, Supplementary Material). This can be explained by the fact that mucus can trap the agglomerates better than surfactant.

### 3.1. Particle Effect in Models of the Bronchial Part of the Respiratory Tract

Calu-3 cells, EpiAirway™ and MucilAir™ tissues were used as models for the bronchial part and A549 and EpiAlveolar™ tissues with and without macrophages as models for the alveolar part. The models were characterized regarding the stability and ability to react to LPS as an inflammatory stimulus. Evaluation of CNT4003 effects over 14 d corresponded to the recommendation of Behrsing et al. for analysis in EpiAirway™s and MucilAir™ tissues [29].

#### 3.1.1. Calu-3 Cells

No cytotoxicity of CNT4003 was seen up to 50 µg with a viability of 86 ± 15% of untreated controls (Figure S2, Supplementary Material).

**Figure 1:**
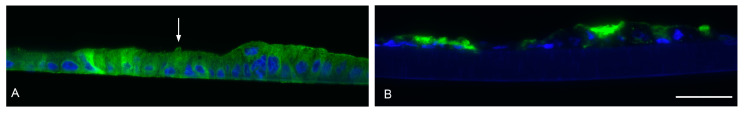
Immunochemical detection of tubulin (**A**) and identification of mucus-producing cells by anti-mucin 5AC staining (**B**). Tubulin is contained in high amounts in cilia and in lower amounts in the cytoskeleton. In the absence of cilia, the staining of the cytoskeleton and of immature cilia (arrow) is visible. Scale bar: 50 µm.

As seen in the immunocytochemical staining, Calu-3 monolayers do not form cilia but contain mucus-producing cells (Figure 1). Alpha tubulin is a microtubule marker, and the tubulin-dynein system is a central part of flagellar and ciliary movement [30]. To a lesser extent, tubulin is also expressed in the cytoplasm for structural support, a pathway for transport and force generation in cell division [31].

**Figure 2:**
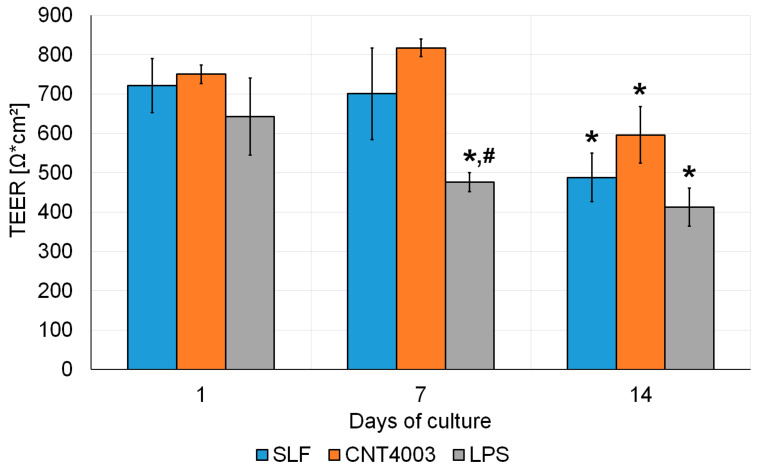
Time-dependent changes of transepithelial electrical resistance (TEER) in Calu-3 cells exposed to simulated lung fluid (SLF) alone, LPS or SLF containing CNT4003. Significant changes compared to 1 d are indicated by an asterisk and significant differences between treated and control cells by a hash.

Stability of the Calu-3 model has been shown previously [32]. In this study, TEER values decreased significantly from 1 d to 14 d of culture in all cultures with no difference between untreated and CNT4003-treated cultures (Figure 2). The decrease upon LPS treatment at 7 d was significant compared to 1 d and also significantly more pronounced than that of the control.

Stimulation with 10 µg LPS increased IL-6 and IL-8 secretions at all time points significantly (Table 1). Interleukin-6 levels of cultures treated with 25 µg CNT4003 after one and seven days were significantly lower than the levels of the controls (Table 1). Interleukin 8 levels were significantly increased after one day and decreased after seven days. No changes were seen after 14 days.

#### 3.1.2. EpiAirway™ Tissues

**Figure 3:**
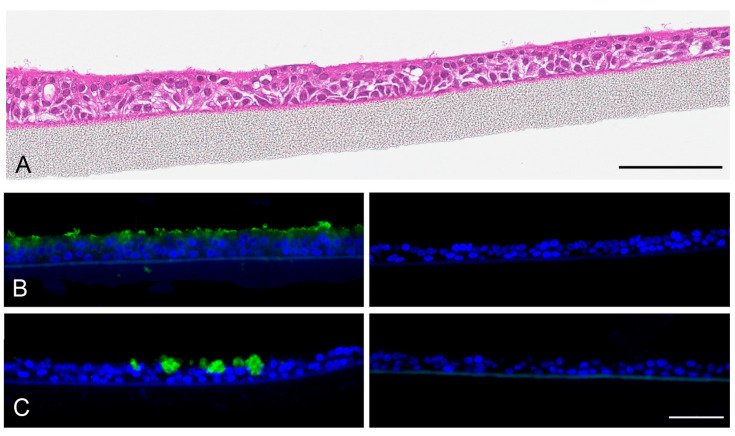
Morphology of EpiAirway™ tissues according to hemalaun staining (**A**) and cellular composition according to immunochistochemical staining. Scale bar: 100 µm. Anti-*α*-tubulin (**B**) and anti-mucin 5AC (**C**) staining (green) for visualization of bronchial epithelial and goblet cells. Controls with mouse IgG instead of primary antibody are shown on the right side. Nuclei are counterstained with Hoechst 33342 (blue). Scale bar 50 µm.

EpiAirway™ cultures are composed of columnar epithelium cells arranged as three to five rows of nuclei (Figure 3A). Cilia at the apical surface are seen at low density. The composition of the epithelium of bronchial epithelial cells and goblet cells is identified by anti-*α*-tubulin staining (Figure 3B) and anti-mucin 5AC staining (Figure 3C), respectively.

TEER values of EpiAirway™ tissues over 28 d were lower than in the other models of the bronchial tract but remained constant for 3 weeks with 259 ± 124 Ω × cm^2^ at 1 d and 244 ± 69 Ω × cm^2^ at 21 d. Only after 28 d, a significant decline to 110 ± 67 Ω × cm^2^ was seen.

**Figure 4:**
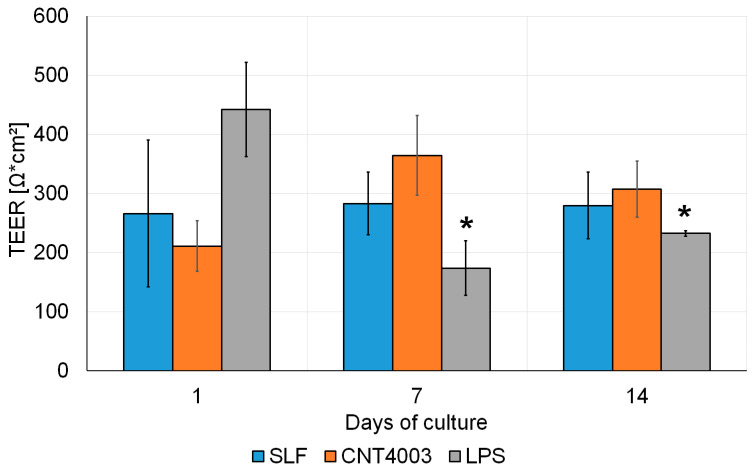
Time-dependent changes of transepithelial electrical resistance (TEER) in EpiAirway™ tissues exposed to simulated lung fluid (SLF) alone or SLF containing CNT4003. Significant decreases compared to 1d are indicated by an asterisk.

CNT4003 particles did not affect TEER values over the entire observation time of 14 d, whereas the initially higher TEER values of the cultures exposed to LPS were significantly decreased compared to 1 d at 7 d and 14 d (Figure 4).

EpiAirway™ tissues reacted to stimulation with all concentrations of LPS with signifiant increases of IL-6 and IL-8 secretions compared to unstimulated tissues at all time points with the exception of IL-6 at day 7 (Table 2).

For assessment of the clearance function of the bronchial epithelium, mucociliary clearance and CBF were evaluated. No transport of the polystyrene indicator beads was detected in the EpiAirway™ tissues, presumably due to the low density of the cilia.

Prior to the assessment of CNT4003, the reaction to LPS was tested. It was found that exposure to LPS (1, 5, 10 µg) increased CBF at all concentrations significantly from 9.9 ± 1.7 Hz (untreated controls) to 15.3–17.8 Hz at 7 d and from 12.7 ± 1.4 Hz (untreated controls) to 15.1–16.2 Hz at 14 d. Basal CBF was significantly lower in this batch of tissues than the frequencies measured in the batch where CNT4003 were tested and no LPS was included. 

CBF of untreated tissues was highest at 1 d and significantly lower at all time points (Table 3). The positive control propranolol decreased CBF significantly at all time points. Significant decreases of CBF upon exposure to CNT4003 compared to the respective medium control were not noted. There was a trend of higher CBF in the peripheral regions (15.8 ± 4.2 Hz) than in the central regions of the insert (14.7 ± 2.8 Hz).

#### 3.1.3. MucilAir™

**Figure 5:**
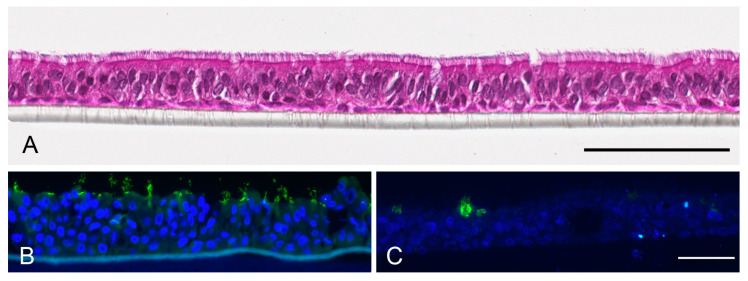
Morphology of MucilAir™ tissues according to hemalaun staining (**A**, scale bar: 100 µm) and cellular composition of the tissues according to immunocytochemistry (**B**,**C**). Anti-*α*-tubulin staining (green, (**B**)) visualized cilia of bronchial epithelial cells and anti-mucin 5AC staining (green, (**C**)) visualized goblet cells. Nuclei are counterstained with Hoechst 33342 (blue). Scale bar 50 µm.

MucilAir™ tissues are composed of two to three rows of cells. Cilia are already visible in the hemalaun staining (Figure 5A). They form much longer structures at the apical surface (Figure 5B) than in the EpiAirway™ tissue (Figure 3B). Further, rare mucin-producing cells can be seen in the MucilAir™ tissues (Figure 5C).

**Figure 6:**
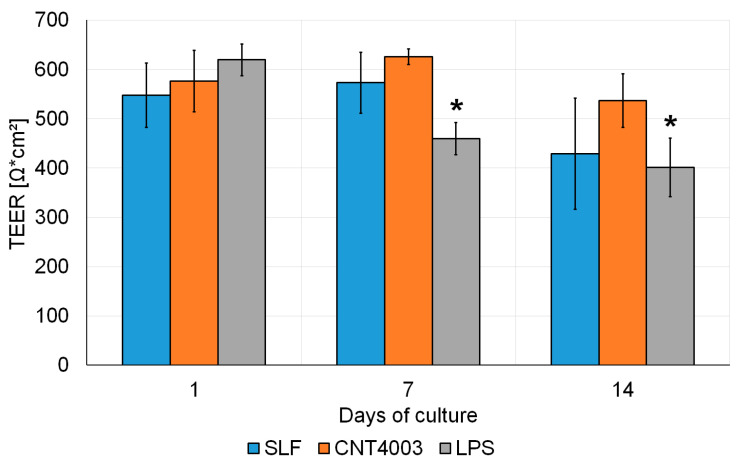
Time-dependent changes in TEER values of MucilAir™ tissues exposed to simulated lung fluid (SLF) alone or with SLF containing CNT4003. Significant decreases compared to 1 d are indicated by an asterisk.

TEER values of the untreated MucilAir™ cultures significantly decreased over 21 d for cultures from 563 ± 66 Ω × cm^2^ at 1 d to 282 ± 24 Ω × cm^2^ at 21 d. Therefore, tissues were studied for only 14 d of exposure with LPS and CNT4003. While TEER values of the LPS-treated tissues were significantly decreased compared to 1 d, CNT4003 particles did not affect TEER values over the entire observation time of 14 d (Figure 6).

The reactivity of the tissues to an inflammatory stimulus was tested by incubation with 1, 5 and 10 µg LPS at 1 d, 7 d and 14 d and quantification of IL-6 and IL-8 was performed. IL-6 levels in LPS-stimulated cultures at all time points and all LPS concentrations were significantly lower than that of the untreated controls (Table 4), which is consistent with other studies reporting low IL-6 levels in LPS-stimulated MucilAir™ tissues. In contrast to the action of IL-6, a robust increase in IL-8 secretion was seen after stimulation with LPS at all concentrations. Metz et al. suggested that concentrations of 10 µg/mL LPS were too low to induce a significant increase in IL-6 [33]. IL-6 secretions of the CNT4003-stimulated MucilAir™ tissues were significantly lower than the unstimulated tissues at all time points (Table 4). IL-8 secretions were also significantly lower than that of the unstimulated controls, except at 1 d.

MucilAir™ tissues were able to transport the polystyrene marker beads. However, since there was no coordinated beating like in the in vivo situation, there was no linear transport of the beads. If transport was seen, the beads moved in circles (Video S1, Supplementary Material). Quantification of the effect of the CNT4003 particles was not possible because the diameter and number of the vortexes showed prominent variations between the tissues. The diameter had an influence on the velocity because transport at the periphery of the vortex was faster than at the center.

CBF of untreated tissues was significantly increased at 14 d compared to 1 d (Table 5). Propranolol treatment decreased CBF significantly at all time points. Additionally, the treatment with 10 µg LPS decreased CBF significantly at 14 d. There was no difference between central (19.5 ± 7.1 Hz) and peripheral regions of the insert (20.6 ± 6.0 Hz). CNT4003 exposure did not changes CBF to significant degree.

### 3.2. Assessment of Effects in Models for the Alveolar Part of the Respiratory Tract

#### 3.2.1. A549 Monocultures and Coculture with THP-1 Macrophages

Exposure to 12.5 and 25 µg CNT4003 had no negative effect on the viability of A549 cells in monoculture and coculture with THP-1, but exposure to 50 µg CNT decreased viability significantly in both types of cultures (Figure S3, Supplementary Material).

A549 cells in mono and coculture with THP-1 macrophages secrete low basal levels of IL-6 (A549 monoculture; 0–1.4 pg/mL and 0.04–0.1 pg/mL in coculture; ref. [27]) and were below the limit of detection in this study. They react, however, at lower concentrations than the bronchial models to LPS and, upon stimulation with LPS, IL-6 levels between 140 and 279 pg/mL in A549 monocultures and between 2 and 3 ng/mL in cocultures with THP-1 were measured. IL-8 levels were 21–27 ng/mL and 53–84 ng/mL, respectively (Table 6). This indicates that the cocultures produced approximately 10 times higher amounts of IL-6 and 4 times higher levels of IL-8 upon LPS stimulation than the A549 monocultures. In contrast to LPS, exposure to 25 µg CNT4003 did not stimulate the secretion of IL-6 and IL-8, which remained below the detection limit.

#### 3.2.2. EpiAlveolar™ Tissues with and without THP-1 Macrophages

**Figure 7:**
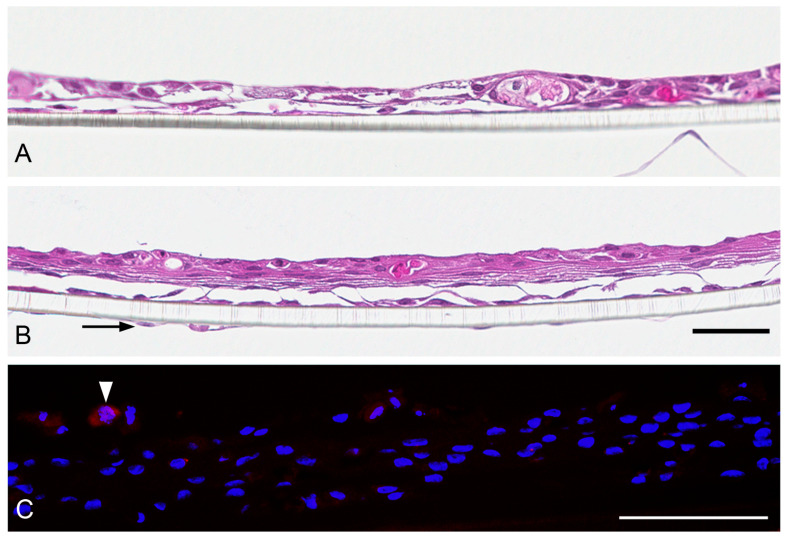
Radial section of EpiAlveolar™ tissues without THP-1 (**A**) and with THP-1 (**B**) macrophages. Endothelial cells at the basal side of the transwell membrane are visible in B (arrow). (**C**) CD45-immunoreactive THP-1 macrophages are rarely seen (arrowhead). Scale bar 50 µm.

EpiAlveolar™ tissues consist of alveolar epithelial cells and fibroblasts at the apical site and endothelial cells at the basal site of the membrane [17]. In the hemalaun-stained sections, three to four rows of cells can be seen and tissues with THP-1 (Figure 7B) appear thicker than those without THP-1 (Figure 7A). In tissues with THP-1 cells, the macrophages were identified by CD45-immunoreactivity (Figure 7C).

Exposure to 25 µg CNT4003 particles did not decrease viability in EpiAlveolar™ tissues with and without THP-1 cells (Figure S4, Supplementary Material).

**Figure 8:**
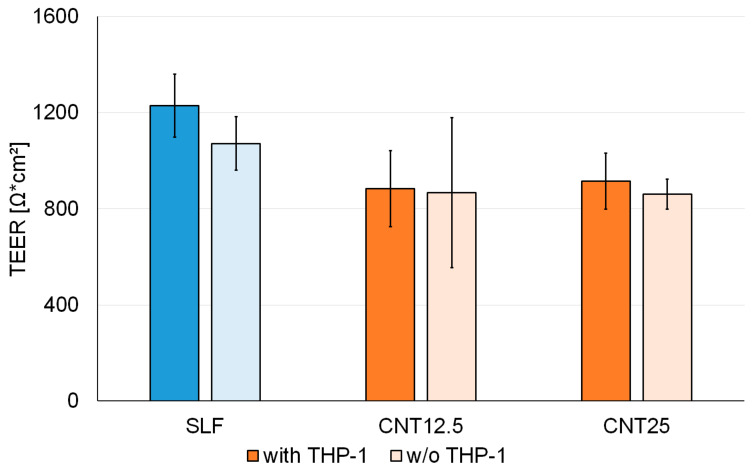
TEER values of EpiAlveolar™ tissues with and without THP-1 treated with control (SLF) or 12.5 and 25 µg CNT4003 particles at 7 d.

EpiAlveolar™ tissues formed tight epithelial barriers with TEER values > 850 Ω × cm^2^ (Figure 8). TEER values of LPS-exposed tissues reached 1260 Ω × cm^2^ in the tissues with THP-1 and 1099 Ω × cm^2^ in the tissues without THP-1. There were no differences between tissues with and without THP-1 macrophages and CNT4003 treatment did not influence barrier tightness. At 14 d, prominent variations between tissue replicates (328–974 Ω × cm^2^ in tissues with THP-1 and 322–1701 Ω × cm^2^ in tissues without THP-1) were noted. TEER values may be a good parameter to screen the quality of the tissues because after one delayed delivery, TEER values of the tissues were 97 ± 137 Ω × cm^2^.

EpiAlveolar™ tissues reacted to stimulation with 1–10 µg LPS with variable and moderate increases in IL-6 and IL-8 secretion. Significant increases in cytokine secretion were mainly seen after stimulation with 5 and 10 µg LPS (Supplementary Material Table S1). In general, more stimulations with significant increases in IL-6 and IL-8 were seen in tissues without THP-1. Treatment with CNT4003 induced a significant increase in IL-6 secretion in EpiAlveolar™ tissues with and without THP-1 macrophages (Table 7). The response in the tissues without THP-1 macrophages was significantly higher than in the tissues with THP-1 cells.

## 4. Discussion

The tightness of the epithelial barrier and low basal levels of proinflammatory cytokines, are crucial properties of the healthy lung, and the used models should be able to assess these parameters. In this comprehensive study, CNTs were evaluated for potential adverse effects on the bronchial and alveolar part of the respiratory system. Further, not only acute cytotoxicity but also epithelial barrier properties, release of cytokines and CBF were used as readout parameters.

### 4.1. Bronchial Models

The suitability of Calu-3 cells as a model for the respiratory barrier and identification of inflammatory effects has already been reported. In this study, TEER values were 210–365 Ω × cm^2^, which is within the range (~100 Ω × cm^2^, ~250 Ω × cm^2^ and 390 Ω × cm^2^) reported in the literature [34,35,36]. Variations in TEER values of EpiAirway™ tissues can show prominent interdonor variations from ~300 Ω × cm^2^ to >900 Ω × cm^2^ [34] and values obtained in this study were at the lower end of this range. MucilAir™ tissues in this study showed initial TEER values of 548 ± 65 Ω × cm^2^. Similar to the EpiAirway™ tissues, there were prominent variations depending on the batch ranging from 346 ± 14 to 638 ± 81 Ω × cm^2^ [37]. The difference between different batches of EpiAirway™ tissues suggests differences between donors or prolonged delivery conditions. 

Similar to the values in this study, IL-6 and IL-8 values in EpiAirway™ tissues were reported as 100–350 pg/mL and 14,500 ± 3000 pg/mL, respectively [8], and 91 ± 40 pg/mL IL-6 and 5300 ± 220 pg/mL IL-8 [36]. Interbatch levels of IL-6 and IL-8 secretion in MucilAir™ can vary by a factor of two to four (IL-6: 58 ± 26–205 ± 130 pg/mL and IL-8: 4.14 ± 0.61–8.19 ± 3.29 ng/mL) [37]. MucilAir™ tissues in this study secreted up to 358 pg/mL IL-6, while IL-8 were around 100 times higher. The much lower IL-6 than IL-8 secretion by MucilAir™ tissues has also been observed in other studies (100 pg/mL vs. 10,000 pg/mL; [38]). Therefore, most researchers used only IL-8 with basal secretions of ~20 ng/mL, 1.8–5.2 ng/mL and 5–30 ng/mL as indicator for inflammation [39,40,41].

Normal CBF in human bronchi was reported as 12–15 Hz [42], but great variation from 4 to 19 Hz were reported for human conducting airways [43]. For bronchial epithelial cells of EpiAirway™, a basal CBF of ~18 Hz (17.62–18.02 Hz) has been reported [44], which is slightly lower than the frequencies determined in this study. MucilAir™ tissues in this study had higher CBF than EpiAirway™ tissues. The different ratio of cilia-bearing to mucus-producing cells between the tissues may be the reason for this difference. The average CBF of MucilAir™ tissues in the literature are indicated as 15.8 ± 0.3 Hz and 11.7 ± 1.2 Hz [43,45], and Beyeler et al. observed higher CBF in the peripheral than in the central region [18]. This trend was also to some extent seen in the EpiAirway™ tissues but not in the MucilAir™ tissues of this study. It indicates that it might be good to use similar regions of the insert for the analysis and indicate that in the protocol, e.g., Kim et al. measured 1–2 mm away from the center of the insert [46]. While propranolol reduced CBF, no marked increase in CBF upon administration of forskolin to the tissues was seen. The lack of a strong increase of CBF was also observed in mouse and rat lung slices [47,48]. The authors hypothesized that cells were preactivated by the mechanical manipulation and beat at their maximum frequency. A decrease of CBF has been observed as a reaction to bacteria and diesel exhaust particles and can be interpreted as a toxic response [42]. In this study, 10 µg LPS decreased CBF in the MucilAir™ tissues but increased it in EpiAirway™ tissues. The different reaction may be due to the different basal CBF, which were ~10 Hz in EpiAirway™ and 28 Hz in MucilAir™ tissues. While the reconstructed tissues presented the advantage that detection of CBF was possible, artificial stimulation of CBF by manual manipulation of the insert, differences due to interdonor differences or damage by long shipping times are disadvantages compared to in-house models based on cell lines.

### 4.2. Alveolar Models

The suitability of A549 cells in mono and in coculture with THP-1 macrophages for the assessment of acute and prolonged effects of particles was shown previously [27]. Assessment of the effect on TEER values is, however, only possible in the reconstructed tissues. Similar to EpiAirway™ and MucilAir™ tissues, marked differences in TEER values were reported for EpiAlveolar™ tissues. We obtained TEER values of 1229 ± 51 Ω × cm^2^ for the EpiAlveolar™ tissues with THP-1 and 1071 ± 111 Ω × cm^2^ for EpiAlveolar™ tissues without THP-1 at 7 d. These data are within the range of the reported values and indicate intact barrier properties. In addition to differences in equipment and operations for the measurements, the shipment of the tissues may impair their viabilities resulting in decreased TEER values compared to those at the producer. TEER values at 7 d and 14 d in the laboratory of the producer were ~1400–1000 Ω × cm^2^ and in another laboratory ~400–600 Ω × cm^2^ [17].

IL-6 levels released by EpiAlveolar™ tissues were reported to vary between 500 pg/mL and 3000 pg/mL in the tissues with THP-1 and between 1500 and 2500 pg/mL in the tissues without THP-1 macrophages [17]. Levels of secreted IL-8 measured over 21 days have been reported as ~5000–15,000 pg/mL in the tissues with THP-1 macrophages and as ~20,000 pg/mL in the tissues without THP-1 macrophages. In this study, IL-6 and IL-8 secretions were in the same order of magnitude as data published by Barosova et al. (e.g., 214 pg/mL–1166 pg/mL in the tissues with THP-1 and 164–1944 pg/mL in those without THP-1 for IL-6 and 5600 pg/mL–26,083 pg/mL in the tissues with THP-1 and from 7476–30,524 pg/mL in EpiAlveolar™ tissues without THP-1 for IL-8). Similar to that study, we did not detect a major influence of THP-1 macrophages on the basal secretion of the interleukins. Upon LPS stimulation, increases were in a similar range for EpiAlveolar™ tissues with and without THP-1 for IL-6 and IL-8 secretions. Coculture with THP-1, by contrast, increased cytokine release markedly upon stimulation with LPS. Increased release of a various proinflammatory cytokines upon LPS stimulation by A549/THP-1 cocultures compared to A549 monocultures has been reported and was reported to be caused by activation of NF-κB [49,50]. The lack of a pronounced response in the EpiAlveolar™ tissues may be due to anti-inflammatory effects by the other cells present in the tissue construct.

### 4.3. Effects of CNT4003

All models for the bronchial tract were stable over 14 d of evaluation and reacted to the proinflammatory stimulus LPS with significant cytokine increases. They were, therefore, regarded as suitable to detect potential toxic effects of CNT4003. The administered dose was 2 × 25 µg CNT4003, which is in the range of the lifetime lung exposure of workers to CNTs (12.4–46.5 µg/cm^2^ lung surface [51]). Exposure to CNT4003 particles caused no adverse effects on viability, epithelial barrier integrity in bronchial models and inflammation. The significant decrease of IL-6 levels in Calu-3 and MucilAir™ tissues might be explained by the anti-inflammatory action of DPPC bound to the CNTs [52]. Lack of effects on cytotoxicity, oxidative stress generation and inflammation was also reported after cumulative (5 × 10 µg/cm^2^) MWCNT exposure of human lung explants [19]. They observed, however, a small increase in CBF from ~8 to ~10 Hz, which was not seen in this study. One explanation may be that the high basal CBF made a further increase impossible. The model, on the other hand, reacted to LPS exposure with a decrease in CBF, indicating that it is capable of identifying adverse effects. We observed in this study that the distribution of the CNT4003 on the insert was not homogenous (Figure S1, Supplementary Material), and one group reported differences in CBF between central and peripheral areas of the insert [18]. Also due to differences in the deposition of the CNTs (Figure S1, Supplementary material) and the fact that not the entire tissue can be evaluated, small local effects may be overlooked.

The two models for the alveolar part of the respiratory tract indicated adverse effects only in the EpiAlveolar™ tissues with and without THP-1 macrophages by increased IL-6 secretion upon exposure to 12.5 and 25 µg CNT4003 with no dose dependency. The greater cytokine release induced by CNT4003 in the EpiAlveolar™ tissues than in the A549 model is in contrast to the more pronounced response of the A549 model to LPS. Greater toxicity of SDS in A549 than in MucilAir™ tissues has been reported and can be explained by the lack of a mucus layer [37]. Protection of the epithelial cell by greater surface coating may also explain the greater sensitivity of the EpiAlveolar™ tissues to CNT4003 particles. It was reported that the surface tension, as an indication for surfactant production, of EpiAlveolar™ tissues was markedly lower than that of A549 cells [17]. Surfactant is produced by alveolar epithelial type II cells, which represent only a small fraction of the EpiAlveolar™ tissues, whereas the A549 cells resemble these cells and are capable to produce surfactant [13].

## 5. Conclusions

Cytotoxicity, TEER values, interleukin secretion and CBF show no indication of adverse effects of CNT4003 in the bronchial part of the respiratory tract. The increased IL-6 secretion of the EpiAlveolar™ tissues may indicate adverse effects of CNT4003 on the alveolar part of the lung. Since the doses for whole-life exposure of workers were applied over a shorter time, the physiological relevance is not clear. Although the reconstructed tissues are produced in a standardized way, considerable variations in basal parameters between batches were seen. In addition to the interdonor differences, the duration of the delivery from the producer to the laboratory of the user has an effect on the viability and reaction of the tissues.

## Figures and Tables

**Table 1:** Time-dependent changes (%) of cytokine secretion by Calu-3 upon stimulation with 10 µg LPS or 25 µg CNT4003. Secretion of unstimulated tissues is set as 100%. Significant differences (*p* < 0.05) between treated and untreated samples are indicated by an asterisk.

Cytokine		Day 1	Day 7	Day 14
Interleukin 6	LPS	478 ± 71 *	703 ± 9 *	722 ± 97 *
Interleukin 8	LPS	1504 ± 69 *	367 ± 88 *	266 ± 32 *
Interleukin 6	CNT4003	55 ± 38 *	25 ± 38 *	116 ± 36
Interleukin 8	CNT4003	118 ± 6*	32 ± 4 *	109 ± 2

**Table 2:** Time-dependent changes (%) of cytokine secretion by EpiAirway™ tissues upon stimulation to 1, 5 and 10 µg lipopolysaccharide (LPS). Secretion of unstimulated tissues is set as 100%. Significant differences (*p* < 0.05) between treated and untreated samples are indicated by an asterisk.

Cytokine	LPS	Day 1	Day 7	Day 14
Interleukin 6	1 µg	228 ± 83 *	89 ± 7	325 ± 18 *
	5 µg	368 ± 165 *	163 ± 7 *	211 ± 18 *
	10 µg	847 ± 327 *	240 ± 12 *	383 ± 24 *
Interleukin 8	1 µg	133 ± 10 *	255 ± 46 *	291 ± 37 *
	5 µg	298 ± 18 *	262 ± 22 *	329 ± 26 *
	10 µg	509 ± 12 *	595 ± 7 *	428 ± 4 *

**Table 3:** Ciliary beating frequency (Hz) in EpiAirway™ tissues after exposure to negative control, positive control (propranolol) or CNT4003. Significant differences (*p* < 0.05) between treated and untreated samples are indicated by an asterisk. Significant changes over time of the untreated tissues are marked by a hash.

Treatment	Day 1	Day 7	Day 14
Medium	34 ± 6	27 ± 2 ^#^	23 ± 8 ^#^
Propranolol	4 ± 0 *	1 ± 0 *	0 ± 0 *
CNT4003	23 ± 10	22 ± 4	16 ± 2

**Table 4:** Time-dependent changes (%) of cytokine secretion by MucilAir™ tissues upon stimulation with 1, 5 and 10 µg lipopolysaccharide (LPS) or CNT4003. Secretion of unstimulated tissues is set as 100%. Significant differences (*p* < 0.05) between treated and untreated samples are indicated by an asterisk. Abbreviation: n.a., not available.

Cytokine	Stimulus	Day 1	Day 7	Day 14
Interleukin 6	LPS_1 µg	69 ± 2 *	n.a. ^a^	44 ± 3 *
	LPS_5 µg	77 ± 17 *	n.a. ^a^	74 ± 15 *
	LPS_10 µg	52 ± 3 *	n.a. ^a^	83 ± 5 *
Interleukin 8	LPS_1 µg	164 ± 2 *	214 ± 172 *	250 ± 142 *
	LPS_5 µg	143 ± 2 *	470 ± 131 *	477 ± 37 *
	LPS_10 µg	277 ± 161 *	443 ± 132 *	659 ± 26 *
Interleukin 6	CNT4003	43 ± 42 *	60 ± 26 *	78 ± 26 *
Interleukin 8	CNT4003	54 ± 12 *	108 ± 13	80 ± 17

^a^ values deleted due to assay interference.

**Table 5:** Ciliary beating frequency (Hz) in MucilAir™ tissues after exposure to control, lipopolysaccharide (LPS) or 25 µg CNT4003. Significant (*p* < 0.05) differences in the medium-treated group at different time points are indicated by an asterisk and differences of the treatment to the medium control with a hash.

Treatment	Day 1	Day 7	Day 14
Medium	15 ± 5	21 ± 6	28 ± 3 ^#^
Propranolol	5 ± 1 *	5 ± 1 *	8 ± 4 *
CNT4003	12 ± 2	18 ± 8	23 ± 7
10 µg LPS	14 ± 3	19 ± 6	19 ± 4 *

**Table 6:** Time-dependent changes in interleukin 6 secretion (pg/mL) and interleukin 8 (ng/mL) secretion by A549 mono and A549/THP-1 coculture upon stimulation with 100 ng/mL lipopolysaccharide (LPS). Since cytokine levels of untreated cultures were zero or below zero, normalization to % of control could not be made.

Cytokine	Day 1	Day 7	Day 14
Interleukin 6_A549	140 ± 33	279 ± 91	182 ± 48
Interleukin 8_A549	21 ± 7	27 ± 9	20 ± 2
Interleukin 6_A549/THP-1	2025 ± 152	2137 ± 587	3195 ± 93
Interleukin 8_A549/THP-1	81 ± 11	53 ± 29	84 ± 20

**Table 7:** Time-dependent changes (in %) of cytokine secretion by EpiAlveolar™ tissues without THP-1 (Epi) and with THP-1 (Epi/THP-1) upon stimulation with lipopolysaccharide (LPS) and CNT4003. Secretion of unstimulated tissues is set as 100%. Significant increases (*p* < 0.05) between treated and untreated samples are indicated by an asterisk and significant decreases by a paragraph sign.

Cytokine	LPS_1 µg	LPS_5 µg	LPS_10 µg	CNT_12.5 µg	CNT_25 µg
Interleukin 6_Epi	21 ± 1 ^§^	112 ± 2	174 ± 7 *	750 ± 244 *	618 ± 72 *
Interleukin 8_Epi	102 ± 12	327 ± 36 *	305 ± 41 *	82 ± 42	93 ± 21
Interleukin 6_ Epi/THP-1	65 ± 30	104 ± 30	214 ± 48 *	288 ± 78 *	390 ± 139 *
Interleukin 8_ Epi/THP-1	100 ± 4	107 ± 81	883 ± 29 *	174 ± 53	102 ± 40

## Data Availability

Datasets analyzed or generated during the study are available upon request from the authors.

## Supplementary Materials

The following supporting information can be downloaded at: https://www.mdpi.com/article/10.3390/nano13040682/s1. Figures S1–S4: Distribution of CNT4003 on cell-grown inserts; cytotoxicity of CNT4003 in Calu-3 cells; cytotoxicity of CNT4003 in A549 cells; cytotoxicity of CNT4003 in EpiAlveolar™ tissues; Table S1: LPS-induced cytokine secretion by EpiAlveolar™ tissues; Video S1: Mucociliary clearance in MucilAir™ tissues.
