# Supplementary material for: Assessment of Carbon Nanotubes on Barrier Function, Ciliary Beating Frequency and Cytokine Release in In Vitro Models of the Respiratory Tract
Source: Nanomaterials (Basel). 2023 Feb 9;13(4):682. doi: 10.3390/nano13040682 (PMC9962067; doi:10.3390/nano13040682)
Supplement: Supplementary file 1 [file nanomaterials-13-00682-s001.zip › nanomaterials-2143765 supp.pdf]

Assessment of Carbon Nanotubes on Barrier Function, Ciliary Beating Frequency and Cytokine Release in In Vitro Models of the Respiratory Tract

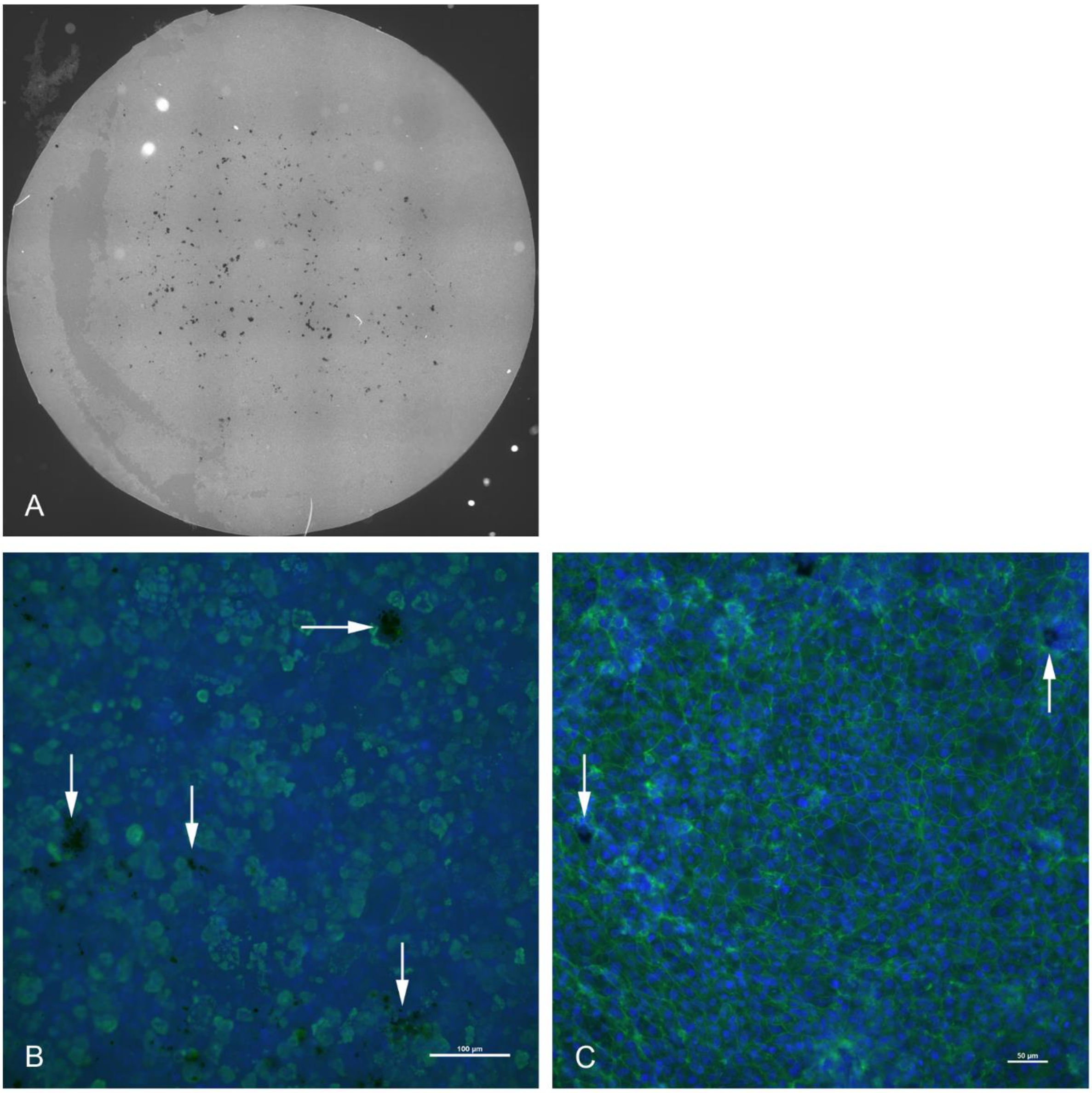

Figure S1. Distribution of CNT4003 on cell-grown inserts. A: More CNT4003 agglomerates are seen in the central region of the inserts. B: on inserts with mucus-producing cells (Calu-3 cells stained with anti-mucin 5AC antibody to show the mucus) more and larger agglomerates are seen than on EpiAlveolar inserts stained with phalloidin to visualize the cytoplasm of the cells (green, C). Scale bar: 100 µm (B), 50 µm (C).

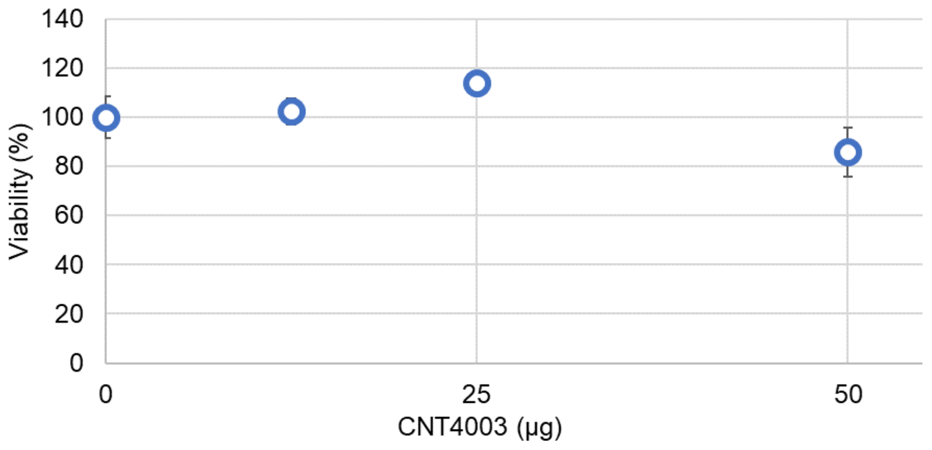

Figure S2. Viability of Calu-3 cells after exposure to CNT4003.

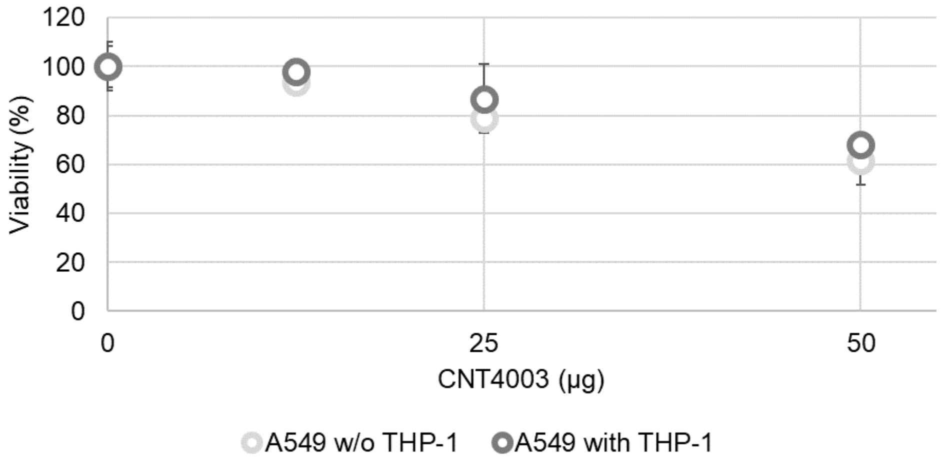

Figure S3. Viability of A549 cells with and w/o THP-1 after exposure to CNT4003.

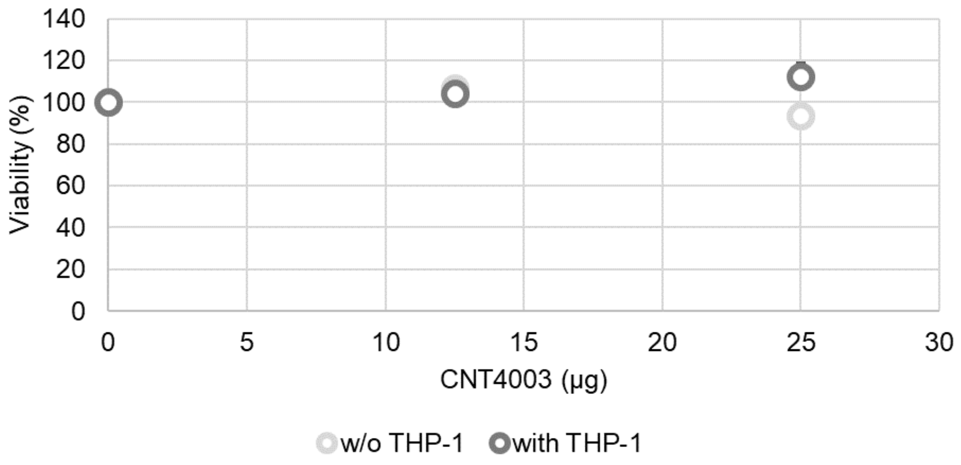

Figure S4. Viability of EpiAlveolar tissues with and w/o THP-1 after exposure to CNT4003.

Table S1. Time-dependent changes (in %) of cytokine secretion by EpiAlveolar™ tissues w/o and with THP-1 upon stimulation with lipopolysaccharide (LPS). Secretion of unstimulated tissues is set as 100%. Significant increases ($p<0.05$) between treated and untreated samples are indicated by asterisk and decreases by paragraph.

Cytokine	LPS	Day 1	Day 7	Day 14
Interleukin 6_w/o THP-1	1μg	50 ± 30	21 ± 1§	115 ± 3
	5 μg	108 ± 4	112 ± 2	147 ± 5*
	10 μg	145 ± 70*	174 ± 7*	129 ± 1*
Interleukin 8_w/o THP-1	1μg	55 ± 17§	102 ± 12	506 ± 36*
	5 μg	151 ± 14*	327 ± 36*	167 ± 8*
	10 μg	162 ± 9*	305 ± 41*	429 ± 1*
Interleukin 6_ with THP-1	1μg	100 ± 6	65 ± 30	160 ± 4*
	5 μg	148 ± 2*	104 ± 30	140 ± 53*
	10 μg	174 ± 10*	214 ± 48*	179 ± 2*
Interleukin 8_with THP-1	1μg	125 ± 13	100 ± 4	116 ± 13
	5 μg	131 ± 16	107 ± 81	126 ± 13*
	10 μg	107 ± 13	883 ± 29*	224 ± 13*
